# Contribution of Food to the Human Health Burden of Antimicrobial Resistance

**DOI:** 10.1089/fpd.2023.0099

**Published:** 2024-02-05

**Authors:** Sonya Kujat Choy, Eva-Marie Neumann, Pablo Romero-Barrios, Sandeep Tamber

**Affiliations:** ^1^ Bureau of Microbial Hazards, Health Products and Food Branch, Health Canada, Ottawa, Canada.; ^2^ Library Services Division, Corporate Services Branch, Health Canada, Ottawa, Canada.

**Keywords:** antimicrobial resistance, foodborne hazard, food safety, risk assessment

## Abstract

The impact of foodborne antimicrobial resistance (AMR) on the human health burden of AMR infections is unknown. The aim of this review was to evaluate and summarize the scientific literature investigating all potential sources of human AMR infections related to food. A literature search was conducted in Embase (Ovid) and MEDLINE (Ovid) databases to identify appropriate studies published between 2010 and 2023. The results of the search were reviewed and categorized based on the primary subject matter. Key concepts from each category are described from the perspective of food safety as a public health objective. The search yielded 3457 references, 1921 remained after removal of duplicates, abstracts, editorials, comments, notes, retractions, and errata. No properly designed source attribution studies were identified, but 383 journal articles were considered relevant and were classified into eight subcategories and discussed in the context of four streams of evidence: prevalence data, epidemiological studies, outbreak investigations and human health impact estimates. There was sufficient evidence to conclude that AMR genes, whether present in pathogenic or nonpathogenic bacteria, constitute a foodborne hazard. The level of consumer risk owing to this hazard cannot be accurately estimated based on the data summarized here. Key gaps in the literature are noted.

## Introduction

**FIG. 1.**
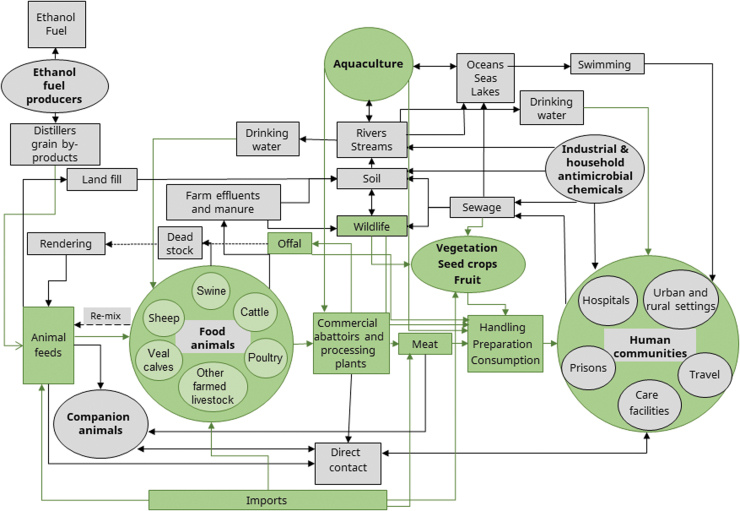
AMR is a One Health issue. Network diagram showing the settings where AMR may emerge, persist, or spread. Bold type indicates areas where antimicrobial use or exposure may occur. Green areas and arrows have a direct link to the foodborne pathway. Adapted from Public Health Agency of Canada (2022). AMR, antimicrobial resistance.

Antimicrobial resistance (AMR) is one of the most significant modern threats to public health (CDC, 2022; WHO, nd). It is a One Health issue, comprising a complex web of interactions across human, animal, and environmental sectors, with multiple routes of transmission and no clear single point of control [Fig. 1; Léger et al. (2021)].

The use of antimicrobials in human medicine and in food production creates selective pressure for an increase in prevalence and spread of antimicrobial-resistant bacteria (ARB) and AMR genes (ARGs) throughout the food supply chain (Collineau et al., 2019; Kenyon, 2021). The spread of AMR through relevant sectors occurs in two ways: the spread of the bacteria that carry resistance genes and the additional spread of resistance genes between bacteria via horizontal gene transfer (HGT). For this reason, it has been suggested that risk analysis should consider the ARGs—rather than the resistant microbe—as the foodborne hazard (Antunes et al., 2020; Wang et al., 2012; Wooldridge, 2012).

Human exposure along the food chain can occur via a wide range of sources and transmission pathways. ARB and/or ARGs have been routinely detected throughout the food production continuum. This includes farms, slaughtering and processing facilities, manufacturing plants, and retail foods. There have also been notable similarities between ARB from food animals and ARB from the humans who handle those food animals.

**FIG. 2.**
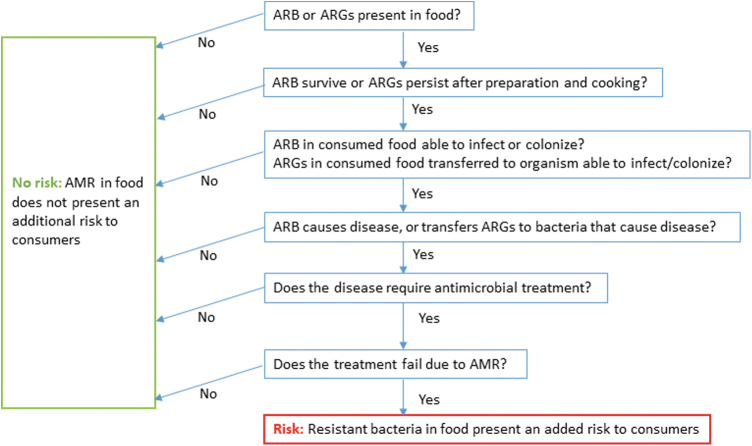
Criteria required for the consideration of AMR as a distinct food safety risk. AMR, antimicrobial resistance; ARB, antimicrobial-resistant bacteria; ARGs, AMR genes.

Current food safety measures implemented by governments, industry, and consumers are expected to be equally effective against antimicrobial-resistant and susceptible pathogens. For foodborne AMR to have a detrimental health impact on consumers, a number of conditions need to be met (Fig. 2). First, the ARB or ARGs need to be present in the food, and they need to survive and persist during food preparation and cooking (if applicable). Once ingested, the ARB should be able to infect or colonize the consumer and cause disease, or the ARG should be transferred to a pathogen that causes disease (either directly, or indirectly via the resident microbiota). Finally, if the disease requires treatment with antimicrobials and those antimicrobials fail to stop the infection because of AMR, we would conclude that the foodborne AMR agent presents a risk to the consumer.

Despite the well-documented presence of resistant microbes and ARGs in the food supply, and the clear pathway for transmission of AMR via food, there is considerable uncertainty regarding the contribution of the foodborne pathway to the AMR problem in humans. We sought to estimate the contribution of foodborne AMR to the human health burden of AMR infections. To address this research question, we conducted a narrative review of the scientific literature with the main goal of identifying source attribution studies that were designed to assess, and quantify, the contribution of foodborne ARB and ARGs to the burden of AMR in humans. We summarized the knowledge that was available, noted the key gaps in the literature, and situated this knowledge within the context of food safety as a public health objective.

## Methods

A search of the scientific literature was conducted to retrieve peer-reviewed articles on AMR resistance and food products. The databases Embase (Ovid) and MEDLINE (Ovid) were searched on June 28, 2021. Searches were restricted to publication dates from January 1, 2010 onward (as it was expected that source attribution studies would be relatively recent), human population, and to English, French and Spanish-language articles. Searches were updated on February 10, 2022 and on January 10, 2023. The results of the database searches were de-duplicated into citation manager RefWorks 2.0 (ProQuest LLC). Detailed database search strategies are presented in the Supplementary Data S1.

We supplemented the database searches with backward citation tracking of the results of the initial database search. Date limits were not applied to the supplemental searches to retrieve background and key studies published before 2010. Final supplemental searches were conducted in April 2022.

The titles and abstracts were reviewed. The inclusion criteria included antibiotics and excluded antiviral and antifungal resistance. Food (products of animal origin, fresh fruit and vegetables, ready-to-eat products) was included, but animals as a reservoir for resistant bacteria or resistance genes were excluded, as our focus was food and not the farm environment. The key concepts were as follows: AMR hazard, food/foodborne, human illness owing to/associated with AMR, incidence of foodborne AMR hazard in the human population (presence or illness), comparison of foodborne versus nonfoodborne AMR in humans, and use of the term “hazard” to refer to AMR microorganism(s) and/or resistance determinant(s) (Codex Alimentarius Commission, 2011). The publications were sorted into categories as detailed in the Results section and Tables 1 and 2.

**Table 1.** Categorization of the Journal Articles Retrieved by the Database Search

Category	Inclusion criteria	No. of references (%)
1. AMR transmission to humans	Articles made a reference to human infection with AMR bacteria from food, or described a comparative analysis of AMR patterns in food and humans (subdivided in Table 2)	383 (9.9)
2. AMR in food	Articles described the presence or levels of AMR bacteria in food without making connections to human health	1069 (55.6)
3. AMR in primary production	The information about AMR was restricted to the farm or production environment	172 (9.0)
4. Control tools for AMR	Articles described different tools to reduce AMR levels in food (e.g., bacteriophages, plant extracts)	95 (4.9)
5. Not relevant	The articles were not considered relevant for the topic of interest. Common examples were: references to testing methodologies, levels of antibiotic residues or generic papers on AMR that did not offer any new information on risk owing to foodborne AMR	202 (10.5)
Total		1921 (100)

AMR, antimicrobial resistance.

**Table 2.** Subcategorization of the “AMR Transmission to Humans” Journal Articles Retrieved by the Database Search

Subcategory	Inclusion criteria	No. of references (%)
1. Surveillance or monitoring studies	Primary research studies or systematic reviews and/or meta-analyses that report and analyze AMR status and prevalence in samples from the food supply chain, the environment and humans	237 (1.9)
2. Epidemiology	Primary research studies or systematic reviews that report analysis of the distribution and identification of potential risk factors for AMR in foodborne disease	17 (4.4)
3. Investigation of foodborne outbreaks	Analysis of data collected as part of an outbreak investigation. Information on epidemiology, health outcomes and/or virulence genes in addition to AMR phenotype or genotype	26 (6.9)
4. Studies estimating human health impact of foodborne AMR	Risk assessments, modeling, exposure estimates; research articles that most directly address the impact of AMR in the food supply chain on human health outcomes	15 (3.9)
5. Horizontal gene transfer	Transmission of ARGs between bacterial species found within the food and fecal microbiomes; factors that impact HGT	10 (2.6)
6. Specific areas of concern	AMR in probiotics, commensals, opportunistic pathogens, fresh produce, and ready-to-eat foods.	14 (3.6)
7. General review	Background information covering multiple categories	26 (7.3)
8. Low relevance	Research articles with weak or tangential evidence regarding AMR transmission to humans via food. Examples include AMR data solely from clinical cases; outbreak data describing a single human isolate	36 (9.4)
Total		383 (100)

AMR, antimicrobial resistance; ARGs, AMR genes; HGT, horizontal gene transfer.

## Results and Discussion

### Summary of data

The database search yielded 3457 references from the scientific literature, and 1921 journal articles after the removal of duplicates and out-of-scope references. The complete list of articles can be found in a spreadsheet in the Supplementary Data S1. These articles were sorted into five categories as described in Table 1. Of those five categories, Category 1, “AMR Transmission to Humans,” was deemed the most likely to contain information directly relevant to the research question, as it contained the studies with data on AMR in both food and humans. These publications were further sorted into eight subcategories according to the criteria in Table 2.

**FIG. 3.**
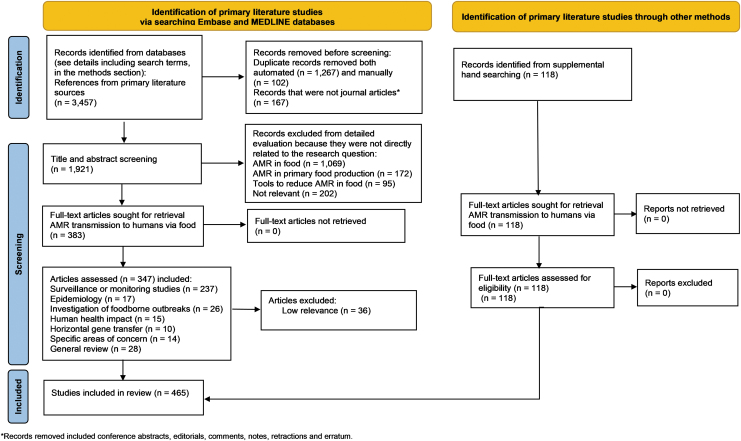
PRISMA diagram describing the literature review and classification process.

In addition to the articles in Tables 1 and 2, the supplemental literature searches resulted in 118 additional references: 47 were considered directly related to the research question, 37 were used to provide support for other data, and 34 were used for background information or context. These searches provided background information or targeted Subcategory 4, “Human Health Impact,” in particular. Figure 3 provides a PRISMA diagram (Page et al., 2021) describing the review and classification process.

Source attribution studies, similar to those conducted for foodborne pathogens [e.g., by the EFSA BIOHAZ Panel (2008)], were considered the ideal form of evidence, but this type of publication was not identified by our search strategy. Rather, four streams of evidence were used to inform our research question (corresponding to Subcategories 1–4 of Table 2: Surveillance or monitoring data, Epidemiological studies, Outbreak investigations, and Human health impact estimates). Some prevalence data were also captured in Subcategories 5 (Table 2: Horizontal gene transfer) and 6 (Table 2: Specific areas of concern). Subcategory 5 described the distribution and movement of ARGs, whereas Subcategory 6 dealt with ARB that are not routinely monitored such as commensals, nonpathogens, and probiotic strains in foods. Together, these two Subcategories (5 and 6) addressed the potential for widespread and unmonitored occurrence of AMR, highlighting emerging or unexpected risks owing to the presence of AMR in the food supply.

### Surveillance or monitoring studies

These studies provided prevalence data and were largely descriptive, documenting the global presence of ARB and/or ARGs in food and humans (Campos et al., 2019; Hong et al., 2016; Khan et al., 2020; Painset et al., 2019; Sodagari et al., 2020). Although some articles implied causality when correlations of specific resistance phenotypes or genotypes were observed between different sampling locations, this type of conclusion should be interpreted with caution given the potential for bias (Cox and Singer, 2012). Neither the individual studies nor meta-analyses of aggregate data were sufficient to support robust conclusions about source attribution and/or the magnitude of the impact of foodborne AMR pathogens on human health outcomes (Cobo-Díaz et al., 2021).

Much of the data in this subcategory was characterized by variability. Some of the variability was inherent to the biology of AMR with differences noted in the frequency and diversity of AMR in foodborne pathogens, as well as the magnitude and mechanism of resistance to specific antimicrobials (Camargo et al., 2017; Hong et al., 2016; Jans et al., 2018). Similarly, the geographic distribution of AMR was variable between and within countries (Cobo-Díaz et al., 2021). As examples, the level of resistance of *Salmonella* in Australia was low compared with the rest of the world (Sodagari et al., 2020), and within Japan the distribution of multidrug-resistant organisms was not uniform across all prefectures (Saito et al., 2020). These differences may have been impacted by culture and food production practices, as well as by geographic location and economic status (Ferrari et al., 2019).

There was uneven coverage and depth of the international data set, potentially reflecting the challenge of collecting and analyzing samples in low- and middle-income countries (Elbediwi et al., 2019; Ronat et al., 2021; Zenebe et al., 2020). Higher quality data were noted when samples were obtained from national or international surveillance repositories because these set out standard operating procedures for collection of samples, analysis, and record-keeping. There was also a focus on meat and food animals, and less comprehensive surveillance of plant-based foods despite their importance as vehicles for food safety hazards (Camargo et al., 2017; Haddadin et al., 2019; Hölzel et al., 2018; Jans et al., 2018).

Methodological variability between individual studies included differences in experimental design, sampling strategy, isolate recovery, antimicrobial susceptibility testing methodology, statistical analysis, and data interpretation. Identification methods ranged from classical, culture-based to molecular, sequence-based and the differences in the sensitivity of detection added to the variability in the datasets and to the difficulty making comparisons between studies. Studies typically used convenience sampling, and different factors that could contribute to bias were not uniformly addressed. For example, potential bias could occur because clinical samples were more likely to be collected from bloodstream infections and these would be more severe compared with common gastrointestinal foodborne disease. Bias could also occur if researchers neglected to use randomized sampling strategies. Furthermore, distinctions were not always made between measurements pertaining to prevalence of ARGs versus ARB, as well as a distinction between AMR prevalence and bacterial load.

The presence of ARGs in commensal bacteria was underrepresented in the search results. Commensals are an important gene reservoir in food because they are widespread, present in higher numbers than pathogens, and regularly consumed via ready-to-eat or uncooked foods. They can carry a large number of ARGs, colonize, and persist in animal and human microbiomes, and participate in HGT (Sparo et al., 2018; Wang et al., 2012). Thus, the presence of ARGs in these microbes is a significantly underestimated foodborne risk [reviewed in Wang et al. (2012)]. Similarly, the microbial communities in fermented foods and probiotics, although not recognized as a risk for AMR in foods, warrant attention owing to variable food safety oversight, high microbial numbers, and as potential reservoirs for ARGs as complex mixtures of undefined microbial species derived from environmental samples (Hachemi et al., 2019; Jans et al., 2018; Rozman et al., 2022; Tóth et al., 2020; Xu et al., 2021).

The data on HGT highlighted the role of ARGs as foodborne hazards and, by extension, the commensals and other nonpathogenic bacteria harboring these genes. Transfer of ARGs occurred between commensal and pathogenic species, in both directions. HGT was documented in many points along the food chain relevant to a foodborne-infection scenario: *in vitro*, *in planta*, in food matrices, and in fecal matter, as well as in *in vitro* and *in vivo* fecal microbiome models (Maeusli et al., 2020; Verraes et al., 2013). If ARGs survive transit through the gastrointestinal tract, these could spread via HGT and could result in persistent AMR in the gut environment (Maeusli et al., 2020). The relevance of HGT for global spread of ARGs was recently illustrated by the widespread presence of the plasmid-borne, previously chromosomal, *mcr-1* gene that confers colistin resistance (Elbediwi et al., 2019).

In addition to overall AMR prevalence, many studies compared the phenotypes and/or genotypes of isolates from different locations or sources, to address whether food could be the source of ARB for consumers. In studies based on a limited number of ARGs, virulence genes and/or phenotypes, the authors often interpreted the data to show similarity between human clinical samples and samples from food or food animals (Abdeen et al., 2021; Dutil et al., 2010; Gormley et al., 2010; Kuang et al., 2014; Overdevest, 2011). By contrast, studies using higher resolution methods based on whole-genome sequencing often revealed divergence between human and foodborne isolates (Acar et al., 2017; Campos et al., 2019; Cummins et al., 2022; Keefer et al., 2019; Lee et al., 2021).

Comparison of different methods for assessing strains—for example pulsed-field gel electrophoresis compared with whole-genome sequencing—suggested that these should yield overall similar results but that the choice of methodology could very well impact conclusions regarding relationships between isolates (Collineau et al., 2019; Signorini et al., 2018).

Regardless of the quality and quantity of prevalence data, the most robust conclusions were that the food supply is a fluctuating reservoir of ARGs, with different prevalence patterns for each resistance phenotype/genotype and bacterial species. Depending on the source of ARGs, the impact on human health can be direct (e.g., treatment failure) or indirect (via HGT), underscoring the importance of defining the hazard *a priori* when assessing the risk of foodborne AMR.

### Epidemiological studies

From the studies focused on AMR transmission via food, 17 of 383 (4.4%) journal articles provided epidemiological data (Table 2: Subcategory 2). This research sought to identify dietary risk factors associated with human AMR infections. The studies included case–control, prospective, cohort, and cross-sectional studies with variable quality of study design and statistical analysis of data. Diet was assessed using surveys, which relied on patient recall and were devoid of information on whether the ARB and/or ARGs were present in the food in question. Clinical outpatient or tertiary care facilities were most frequently used to recruit cases and controls, with or without consideration of underlying or preexisting nosocomial risk factors; and fewer studies used healthy volunteers. In general, the clinical infections were characterized as resistant or susceptible to antimicrobials, thus the results could have been strengthened by comparison with relevant prevalence data on AMR in the appropriate food supply chain, as in the study by Mulder et al. (2019).

Overall, the epidemiological studies provided weak evidence for source attribution to food through identification of possible dietary risk factors in patients with clinical AMR infections. The risk factors were typically either a general food category such as pork (de Lauzanne et al., 2022) or a specific food such as papaya salad (Tongtawee et al., 2016). The results were clearly influenced by geographic and cultural factors and should not be generalized beyond the specific study population. The possibility that different ARGs could be associated with specific dietary risk factors was demonstrated by a large study that collected data on uropathogenic *Escherichia coli* (Mulder et al., 2019). This was a population-based prospective cohort study where the infections were categorized according to resistance phenotype, with analysis of the dietary factors associated with each resistance phenotype.

There was variability between individual studies in strength or direction of association to a particular risk factor; for example, consumption of chicken was identified as a risk factor in one study but as a protective factor for *Campylobacter* infection in another study [compare Hu et al. (2021); Komba et al. (2015)]. The clinical infections tended to be extra-intestinal infections whose direct acquisition via foodborne transmission could not be established (Mulder et al., 2019; van Rijen et al., 2013). Thus, any dietary associations would reflect an indirect route of transmission where the ARG itself would have been the hazard. One study searched for dietary risk factors associated with carriage of ARGs in healthy volunteers, but this study could neither identify nor predict the magnitude of the human health impact for these ARGs (Hu et al., 2021).

The epidemiological studies did not provide evidence that could be used for source attribution of AMR to food, as they were not designed to compare different sources of AMR. In general, they were best suited for revealing risk factors with strong direct association to AMR human health outcomes, such as previous antibiotic treatment (Saito et al., 2020) or use of clinical devices [e.g., catheters; Kalluru et al. (2018)]. By comparison, the dietary risk factors described previously showed relatively weaker associations (de Lauzanne et al., 2022; Hu et al., 2021; Kalluru et al., 2018; Komba et al., 2015; Saito et al., 2020; Tongtawee et al., 2016; van Rijen et al., 2013).

### Investigation of foodborne outbreaks

Twenty-six of 383 journal articles, included in Subcategory 3, AMR transmission via food (6.9%, Table 2), provided data from outbreak investigations. Additional meta-analysis articles were obtained by supplemental literature searches. Whereas proof-of-principle, individual outbreak investigations confirmed that transmission of ARB occurred via the food supply chain and that there was an impact on human health, meta-analyses provided population-level information on excess morbidity and mortality owing to AMR.

The outbreak investigation articles contained sufficient information for assessing the human health impact related to the AMR characteristics of the foodborne pathogen. Of particular value were meta-analyses that compared the human health outcome of outbreaks linked to resistant versus susceptible pathogens. For example, these studies revealed the association of resistant *Salmonella* with higher levels of hospitalization and/or mortality (Parisi et al., 2018; Varma et al., 2005). Excess health impacts attributed to AMR were different between, and dependent upon, the specific bacterial pathogens; for example, *Listeria* outbreaks had more adverse effects than *Salmonella* outbreaks (Mäesaar et al., 2021; Mølbak et al., 1999).

Fresh produce and ready-to-eat foods were as important as meat and meat products as a source of foodborne AMR pathogens (Karlsson et al., 2021), but not all the possible foodborne pathogens were represented in the outbreak investigation reports provided by the database search results. Risk factors related to host susceptibilities in vulnerable populations such as extremes of age and preexisting conditions were often present in outbreak studies that reported mortality (Holmberg et al., 1984; Mølbak et al., 1999).

Outbreak investigations that provided detailed information on bacterial phenotypes and genotypes were able to compare and trace isolates from human clinical samples with isolates from specific food, food animals, and/or food establishments or processing facilities. Typically, in individual outbreak datasets, the bacterial sampling coverage was incomplete, and the identification of the source was supported by additional epidemiological commonalities (Mikhail et al., 2021). Some outbreak investigations reported AMR simply as adjunct information or used it as the microbial genetic signature for source identification, and most articles did not consider the impact of ARGs on bacterial pathogenicity and human health outcomes (Gieraltowski et al., 2016; Hoffmann et al., 2014). Some reports did not separate single drug–resistant from multidrug-resistant infections; others omitted data on the impact of AMR on patient treatment protocols (Hoffmann et al., 2014). Furthermore, some of the more severe clinical cases were only indirectly linked to the presumed food source, for example, via nosocomial or human-to-human transmission (Holmberg et al., 1984; Mølbak et al., 1999).

The database search also returned articles documenting clinical infections owing to opportunistic pathogens where the infection itself was not considered foodborne but where the bacterial agent—and/or its resistance genes—had been found in the food supply chain. This was noted for pseudomonads (Abd El-Ghany, 2021; Raposo et al., 2017), *Klebsiella* (Davis and Price, 2016), *E. coli* (Manges et al., 2007) and methicillin-resistant *Staphylococcus aureus* (EFSA BIOHAZ Panel, 2009). There was a notable lack of consensus regarding the significance of AMR and the strength of the linkage from food to human infection for these pathogens.

### Modeling studies to estimate the human health impact of foodborne AMR

Fifteen of 383 journal articles (3.9%, Table 2) described models for the transmission of ARGs and ARB through food, which were used to generate quantitative exposure estimates and risk assessments. Modeling, risk assessments, and exposure estimates were conducted for specific food–pathogen–AMR combinations (Collineau et al., 2019; Hölzel et al., 2018; Jans et al., 2018). These studies typically integrated data from multiple sources, with a particular reliance upon AMR prevalence reports and surveillance data (Jans et al., 2018; Travers and Barza, 2002; Zhang et al., 2021a). As needed, research groups made assumptions or generated additional data for pathogen growth, spread, and inactivation (e.g., due to cooking) and for population-level data of consumption of particular foods (Snyder et al., 2013; Zhang et al., 2021a).

Based on modeling, most research supported the conclusion that ARB and ARGs are present at sufficient prevalence and concentration to result in consumer exposure, whether through meat-based or produce-based foods (Nekouei et al., 2018; Njage and Buys, 2017; O'Flaherty et al., 2019; Zhang et al., 2021b). In general, the models did not distinguish between resistant and susceptible microorganisms when estimating parameters of bacterial growth, survival, and spread.

The presence of ARB on food at the retail level potentiated their introduction into consumer kitchens, where they could spread via utensils and food preparation surfaces. Not surprisingly, the risk estimates were higher when the contamination was spread to foods eaten without further cooking (Plaza-Rodríguez et al., 2019; Snyder et al., 2013). Food safety measures and hygiene in the home kitchen were considered the most effective means to reduce the risk of consumer exposure (Plaza-Rodríguez et al., 2019; Zhang et al., 2022; Zhang et al., 2021b). Cooking was considered to be effective at inactivating ARB on meat and meat products (Bacon et al., 2003), whereas irrigation water was the critical control point for reducing ARB concentration on fresh produce (Njage and Buys, 2017; O'Flaherty et al., 2019).

A number of studies provided quantitative risk assessment data that support estimates of the human health impact of foodborne AMR. For example, Collineau et al. (2020) estimated the public health risk for consumption of chicken contaminated with ceftiofur-resistant *Salmonella enterica* serotype Heidelberg and assessed the potential impact of several interventions. Withdrawing preventive ceftiofur use in poultry, improving on-farm cleaning regimens, changing chilling practices, and improving storage and preparation of meat were the most effective interventions in their model. An earlier study calculated the quantitative risk for human health because of the use of fluoroquinolones in dairy cattle and estimated that the risk of fluoroquinolone treatment failure in humans when treating foodborne diseases caused by *Campylobacter* and *Salmonella* was very low (measured in cases per billion) (Hurd et al., 2010).

There is growing consumer demand for meat from animals raised without antibiotics, but one study concluded that this type of husbandry may not result in significant changes to consumer exposure to ARB (Zhang et al., 2021b). One study was valuable for suggesting alternative viewpoints on factors that could impact transmission of AMR within the food supply chain; it focused on the use of antibiotics in food animals as a source of AMR in humans and used different assumptions for ARB transmission when modeling the spread of pathogens between human and animal host populations (van Bunnik and Woolhouse, 2017). However, it is significant to note that none of the studies modeled the actual risk of infection, but only of exposure to ARB or ARGs.

The key gap in research data was the lack of dose–response models, a gap that could be linked to the difficulties associated with defining clinical outputs for resistant bacteria. Other research highlighted the difficulties in finding suitable data for detailed farm-to-fork risk assessments that try to follow Codex Guidelines for risk analysis of foodborne AMR (Taylor et al., 2022).

## Conclusions

We did not find source attribution studies quantifying the contribution of food to the burden of AMR in humans but, taken together, the four streams of evidence used in this review were sufficient to conclude that ARB and ARGs, regardless of pathogenicity or host status, constitute a foodborne hazard.

Although there were insufficient data to estimate an overall health risk to consumers owing to food contaminated with AMR determinants, there were examples that demonstrated that the level of risk is higher for vulnerable populations and from the consumption of a subset of foods (e.g., ready-to-eat foods). Because this is true for foodborne illness in general, it is likely that good hygiene and food safety practices would be similarly effective for mitigating the risk of AMR at the point of consumption. As this may not align with consumer perception, risk communication could emphasize the efficacy of food safety practices as a protective measure against AMR in food especially among vulnerable populations in high-risk settings (Ancillotti et al., 2022; Ritter et al., 2019). It is not known whether additional measures, such as microbiological criteria specifically for resistant bacteria, are required. Should these measures be implemented, further work would be needed to define the hazards in terms of resistance type, bacterial species, or ARGs.

In addition to the lack of source attribution studies mentioned previously, each stream of evidence had limitations that highlighted the gaps in our understanding of foodborne AMR (Table 3). Research is needed to provide estimates of the burden of human infections with resistant organisms that can be attributed to food, compared with other sources of AMR. Prevalence data, which is foundational for risk assessments, have higher reliability when aggregated by meta-analysis. Accordingly, these studies should be designed to evaluate the relationship between the presence of resistant bacteria in food and in humans using standardized methods. However, many prevalence studies relied on convenience sampling, and fit-for-purpose methods. Thus, comparative studies should be carefully assessed by readers before accepting the authors' conclusions, and those who publish should apply study designs that avoid bias along with careful interpretation of results to avoid unwarranted statements of causality.

**Table 3.** Research Gaps in Our Knowledge of Foodborne Antimicrobial Resistance

Gap/evidence needed
Fit-for-purpose source attribution studies to provide estimates of the burden of human infections with resistant organisms that can be attributed to food, compared with other sources of AMR
Dose–response models for resistant pathogens; clearly defined clinical outcomes (e.g., infection, illness, or treatment failure)
Expanded AMR surveillance to include underrepresented food sources and all resistant organisms, including nonpathogenic bacteria
More information collected during foodborne outbreak investigations; AMR status of the hazard involved, minimum inhibitory concentration, clinical outcomes affected by resistance
Estimates of occurrence of HGT in food matrices, food production areas, and human gastrointestinal tract
Standardized (comparable) methods for the detection, quantification, AMR phenotyping, and subtyping of ARB, knowledge of method parameters (e.g., sensitivity, specificity)

AMR, antimicrobial resistance; ARB, antimicrobial resistant bacteria; HGT, horizontal gene transfer.

There was convincing evidence for HGT of ARGs. This has several important implications. For example, the movement of ARGs between bacterial hosts needs to be incorporated into the interpretation of prevalence data. Nonpathogenic bacteria, commensals, and opportunistic pathogens are not routinely included in monitoring activities, and are part of the reservoir of ARGs that can enter the food supply chain but that would not necessarily be addressed by food safety protocols. If ARGs are considered as the hazard, effective mitigation of foodborne AMR would need to target a much broader range of bacteria beyond those that are currently monitored. In addition, research and surveillance activities could be expanded to include foods currently underrepresented, such as produce, fermented foods, and ready-to-eat products.

The epidemiological studies demonstrated only weak association between dietary risk factors and AMR infections. These results could have been strengthened by inclusion of prevalence data for AMR determinants in the relevant foods.

The outbreak investigations provided proof-of-principle that transmission of ARB could occur via the food supply chain and result in adverse human health outcomes, including treatment failure. To better understand burden of illness, the AMR status of the hazard and the clinical outcomes affected by resistance (e.g., treatment failures) could be routinely included in the information captured during outbreak investigations. Although there were relatively few systematic review and meta-analysis articles, these provided population-level information on excess morbidity and mortality in outbreaks owing to resistant versus susceptible pathogens. The human health impact research comprised risk assessment and exposure estimate models, relying heavily upon AMR prevalence reports and surveillance data but hindered by the lack of dose–response models.

The lack of dose–response models for resistant pathogens was a key gap in the literature. As a result, many of the risk assessment models arrived at an exposure estimate but could not extend the analyses to calculate the potential risk of adverse human health outcomes. Risk assessments were further complicated by lack of adequate data and the challenge of selecting or defining appropriate clinical outcomes for resistant infections owing to foodborne pathogens (e.g., infection, disease, or treatment failure). Some foodborne infections are self-limiting, others are contraindicated for antibiotic treatment, and severe outcomes such as hospitalization or death might be overrepresented in clinical samples. These factors may therefore introduce bias into the human prevalence data. Additional research is needed to clearly define which clinical outcomes are relevant for risk assessment of foodborne AMR. This would facilitate the development of dose–response models that are currently not available.

In conclusion, the movement of ARB and ARGs through the food supply has been well documented, but challenges in calculating the magnitude of the risk of foodborne AMR to human health remain. Further research is needed to address the current knowledge gaps in source attribution and risk assessment of foodborne AMR. Despite these gaps, action to mitigate foodborne AMR may be warranted in areas where the risk is elevated, such as the food served in hospitals and to vulnerable populations.

## Supplementary Material

Supplemental data
