# Supplementary material for: Contribution of Food to the Human Health Burden of Antimicrobial Resistance
Source: Foodborne Pathog Dis. 2024 Feb 5;21(2):71–82. doi: 10.1089/fpd.2023.0099 (PMC10877391; doi:10.1089/fpd.2023.0099)
Supplement: Supplemental data [file Suppl_DataS1.zip › Kujat.Choy.Supp.Mat.docx]

**DATABASE SEARCH STRATEGIES**

Database(s): **Embase**1974 to 2023 January 11
Search Strategy:

| **#** | **Searches** | **Results** |
| --- | --- | --- |
| 1 | exp *antibiotic resistance/ | 64272 |
| 2 | (AMR or ARGs or MRSA or ((antimicrobial* or microbial* or antibacterial* or antibiotic* or anti biotic* or bacteria* or gene or genes or multidrug* or multi drug* or multiple drug*) adj3 resistan*)).ti. | 75789 |
| 3 | (AMR or ARGs or MRSA or ((antimicrobial* or microbial* or antibacterial* or antibiotic* or anti biotic* or bacteria* or gene or genes or multidrug* or multi drug* or multiple drug*) adj3 resistan*)).ab. /freq=2 | 108002 |
| 4 | ((aldesulfone sodium or amidinopenicillin* or amikacin or aminocyclitol or aminoglycoside* or aminopenicillin* or (aminosalicylate* adj2 calcium) or (aminosalicylate* adj2 sodium) or amoxicillin* or amoxicillin-clavulanicacid or amphenicol? or ampicillin* or azidocillin* or ampicillin-sulbactam? or ansamycin? or antipseudomonal? or antistaphylococcal* or apramycin or arbekacin or astromicin or avoparcin or azithromycin or azlocillin* or aztreonam or bacampicillin* or bacitracin* or bedaquilin? or bekanamycin or benethaminebenzylpenicillin* or benzathinebenzylpenicillin* or benzylpenicillin* or besifloxacin or biapenem or brodimoprim or cadazolid or capreomycin or carbapenem? or carbenicillin* or carindacillin* or carumonam or cefacetrile or cefaclor or cefadroxil or cephalexin? or cefalonium or cefaloridin? or cefalothin* or cefalotin* or cefamandole or cefapirin or cefatrizine or cefazedone or cefazolin or cefbuperazone or cefcapene or cefdinir or cefditoren or cefepime or cefetamet or cefixime or cefmenoxime or cefmetazole or cefminox or cefodizime or cefonicid or cefoperazone or cefoperazone-sulbactam or cefoselis or cefotaxime or cefotetan or cefotiam or cefoxitin or cefovecin or cefozopran or ceforanide or cefpiramide or cefpirome or cefpodoxime or cefprozil or cefquinome or cefradine or cefroxadine or cefsulodin or ceftaroline fosamil or ceftazidime or ceftazidime-avibactam or ceftezole or ceftibuten or ceftiofur or ceftizoxime or ceftobiprole or ceftolozane or ceftriaxone or ceftriaxone-sulbactam or cefuroxime or cephalosporin* or cephamycin* or cethromycin or chloramphenicol or chlortetracycline or cinoxacin or ciprofloxacin or clarithromycin or clindamycin or clofazimine or clometocillin* or clomocycline or cloxacillin* or colistin or cycloserine or dalbavancin or danofloxacin or dapsone or daptomycin or delafloxacin* or delamanid or dibekacin* or dicloxacillin* or difloxacin* or dihydrostreptomycin* or dirithromycin* or demeclocyclin* or dihydrofolate reductase inhibitor* or doripenem or doxycycline or enoxacin or enrofloxacin or epicillin* or eravacycline or ertapenem or erythromycin or ethambutol or ethionamide or faropenem or fidaxomicin or fleroxacin or flumequine or fluoroquinolone? or flurithromycin or flomoxef or florfenicol or flucloxacillin* or formosulfathiazole or fosfomycin or framycetin or furaltadone or furazidin or furazolidone or fusidane or fusidic acid* or gamithromycin or garenoxacin* or gatifloxacin* or gemifloxacin* or gentamicin* or glycopeptide* or glycylcyclin* or grepafloxacin* or hetacillin* or ibafloxacin or iclaprim or imipenem or isepamicin or isoniazid or josamycin or kanamycin or ketolide? or kitasamycin or latamoxef or levofloxacin or lincomycin or lincosamide? or linezolid or lipoglycopeptide? or lipopeptide? or lomefloxacin or loracarbef or lymecycline or macrolide? or marbofloxacin or mecillinam or meropenem or metampicillin* or mezlocillin* or metacycline or methicillin* or meticillin* or metronidazole or midecamycin or minocycline or miocamycin or monobactam* or morinamide or moxifloxacin or mupirocin or nadifloxacin or nafcillin* or nalidixic acid* or naphcillin* or neomycin or netilmicin or nifurtoinol or nitrofuran? or nitrofurantoin or nitrofural or nitroimidazole* or norfloxacin or ofloxacin or oleandomycin* or omadacyclin* or orbifloxacin or oritavancin or ornidazole or oxacillin* or oxazolidinone? or oxolinic acid* or oxytetracycline or ozenoxacin or panipenem or para-aminosalicylic-acid* or paromomycin or pazufloxacin or pefloxacin or penamecillin* or penethamate hydriodide or penicillin? or penimepicycline or pheneticillin* or phenoxymethylpenicillin* or phthalylsulfathiazole or pipemidic acid* or piperacillin* or piperacillin-tazobactam or pirlimycin or piromidic acid* or pivampicillin* or pivmecillinam or plazomicin or pleuromutilin? or polypeptide? or polymyxin? or polymyxin b or polymixin e or pradofloxacin or pristinamycin or procaine or propicillin* or protionamide or prulifloxacin or pseudomonic acid* or pyrazinamide or pyrimethamine or quinolone? or quinupristin-dalfopristin or ramoplanin or radezolid or retapamulin or ribostamycin or rifabutin or rifampicin or rifamycin or rifapentine or rifaximin or riminofenazine* or rokitamycin or rolitetracycline or rosoxacin or roxithromycin or rufloxacin or secnidazole or sitafloxacin or solithromycin or sparfloxacin or spectinomycin or spiramycin or streptogramin? or streptomycin or sulbenicillin* or sulfadiazine or sulfadimethoxine or sulfadimidine or sulfafurazole or sulfaisodimidine or sulfalene or sulfamazone or sulfamerazine or sulfamethizole or sulfamethoxazole or sulfamethoxypyridazine or sulfametomidine or sulfametoxydiazine or sulfametrole or sulfamoxole or sulfanilamide or sulfaperin or sulfaphenazole or sulfapyridine or sulfathiazole or sulfathiourea or sulfisoxazole or sulfonamide? or sulfone or sulfones or sultamicillin* or talampicillin* or tazobactam or tedizolid or teicoplanin or telavancin or telithromycin or temafloxacin or temocillin* or terizidone or tetracycline? or tetroxoprim or thiamphenicol or tiamulin or ticarcillin* or ticarcillin-clavulanicacid or tigecycline or tildipirosin or tilmicosin or tinidazole or tiocarlide or tobramycin or trimethoprim or troleandomycin or tulathromycin or tylvalosin or tylosin or vaborbactam or valnemulin or vancomycin or virginiamycin) adj2 resistan*).ti. | 48464 |
| 5 | ((aldesulfone sodium or amidinopenicillin* or amikacin or aminocyclitol or aminoglycoside* or aminopenicillin* or (aminosalicylate* adj2 calcium) or (aminosalicylate* adj2 sodium) or amoxicillin* or amoxicillin-clavulanicacid or amphenicol? or ampicillin* or azidocillin* or ampicillin-sulbactam? or ansamycin? or antipseudomonal? or antistaphylococcal* or apramycin or arbekacin or astromicin or avoparcin or azithromycin or azlocillin* or aztreonam or bacampicillin* or bacitracin* or bedaquilin? or bekanamycin or benethaminebenzylpenicillin* or benzathinebenzylpenicillin* or benzylpenicillin* or besifloxacin or biapenem or brodimoprim or cadazolid or capreomycin or carbapenem? or carbenicillin* or carindacillin* or carumonam or cefacetrile or cefaclor or cefadroxil or cephalexin? or cefalonium or cefaloridin? or cefalothin* or cefalotin* or cefamandole or cefapirin or cefatrizine or cefazedone or cefazolin or cefbuperazone or cefcapene or cefdinir or cefditoren or cefepime or cefetamet or cefixime or cefmenoxime or cefmetazole or cefminox or cefodizime or cefonicid or cefoperazone or cefoperazone-sulbactam or cefoselis or cefotaxime or cefotetan or cefotiam or cefoxitin or cefovecin or cefozopran or ceforanide or cefpiramide or cefpirome or cefpodoxime or cefprozil or cefquinome or cefradine or cefroxadine or cefsulodin or ceftaroline fosamil or ceftazidime or ceftazidime-avibactam or ceftezole or ceftibuten or ceftiofur or ceftizoxime or ceftobiprole or ceftolozane or ceftriaxone or ceftriaxone-sulbactam or cefuroxime or cephalosporin* or cephamycin* or cethromycin or chloramphenicol or chlortetracycline or cinoxacin or ciprofloxacin or clarithromycin or clindamycin or clofazimine or clometocillin* or clomocycline or cloxacillin* or colistin or cycloserine or dalbavancin or danofloxacin or dapsone or daptomycin or delafloxacin* or delamanid or dibekacin* or dicloxacillin* or difloxacin* or dihydrostreptomycin* or dirithromycin* or demeclocyclin* or dihydrofolate reductase inhibitor* or doripenem or doxycycline or enoxacin or enrofloxacin or epicillin* or eravacycline or ertapenem or erythromycin or ethambutol or ethionamide or faropenem or fidaxomicin or fleroxacin or flumequine or fluoroquinolone? or flurithromycin or flomoxef or florfenicol or flucloxacillin* or formosulfathiazole or fosfomycin or framycetin or furaltadone or furazidin or furazolidone or fusidane or fusidic acid* or gamithromycin or garenoxacin* or gatifloxacin* or gemifloxacin* or gentamicin* or glycopeptide* or glycylcyclin* or grepafloxacin* or hetacillin* or ibafloxacin or iclaprim or imipenem or isepamicin or isoniazid or josamycin or kanamycin or ketolide? or kitasamycin or latamoxef or levofloxacin or lincomycin or lincosamide? or linezolid or lipoglycopeptide? or lipopeptide? or lomefloxacin or loracarbef or lymecycline or macrolide? or marbofloxacin or mecillinam or meropenem or metampicillin* or mezlocillin* or metacycline or methicillin* or meticillin* or metronidazole or midecamycin or minocycline or miocamycin or monobactam* or morinamide or moxifloxacin or mupirocin or nadifloxacin or nafcillin* or nalidixic acid* or naphcillin* or neomycin or netilmicin or nifurtoinol or nitrofuran? or nitrofurantoin or nitrofural or nitroimidazole* or norfloxacin or ofloxacin or oleandomycin* or omadacyclin* or orbifloxacin or oritavancin or ornidazole or oxacillin* or oxazolidinone? or oxolinic acid* or oxytetracycline or ozenoxacin or panipenem or para-aminosalicylic-acid* or paromomycin or pazufloxacin or pefloxacin or penamecillin* or penethamate hydriodide or penicillin? or penimepicycline or pheneticillin* or phenoxymethylpenicillin* or phthalylsulfathiazole or pipemidic acid* or piperacillin* or piperacillin-tazobactam or pirlimycin or piromidic acid* or pivampicillin* or pivmecillinam or plazomicin or pleuromutilin? or polypeptide? or polymyxin? or polymyxin b or polymixin e or pradofloxacin or pristinamycin or procaine or propicillin* or protionamide or prulifloxacin or pseudomonic acid* or pyrazinamide or pyrimethamine or quinolone? or quinupristin-dalfopristin or ramoplanin or radezolid or retapamulin or ribostamycin or rifabutin or rifampicin or rifamycin or rifapentine or rifaximin or riminofenazine* or rokitamycin or rolitetracycline or rosoxacin or roxithromycin or rufloxacin or secnidazole or sitafloxacin or solithromycin or sparfloxacin or spectinomycin or spiramycin or streptogramin? or streptomycin or sulbenicillin* or sulfadiazine or sulfadimethoxine or sulfadimidine or sulfafurazole or sulfaisodimidine or sulfalene or sulfamazone or sulfamerazine or sulfamethizole or sulfamethoxazole or sulfamethoxypyridazine or sulfametomidine or sulfametoxydiazine or sulfametrole or sulfamoxole or sulfanilamide or sulfaperin or sulfaphenazole or sulfapyridine or sulfathiazole or sulfathiourea or sulfisoxazole or sulfonamide? or sulfone or sulfones or sultamicillin* or talampicillin* or tazobactam or tedizolid or teicoplanin or telavancin or telithromycin or temafloxacin or temocillin* or terizidone or tetracycline? or tetroxoprim or thiamphenicol or tiamulin or ticarcillin* or ticarcillin-clavulanicacid or tigecycline or tildipirosin or tilmicosin or tinidazole or tiocarlide or tobramycin or trimethoprim or troleandomycin or tulathromycin or tylvalosin or tylosin or vaborbactam or valnemulin or vancomycin or virginiamycin) adj2 resistan*).ab. /freq=2 | 52956 |
| 6 | 1 or 2 or 3 or 4 or 5 [AMR] | 204670 |
| 7 | ("animal-based food*" or "food? of animal origin*" or "product? of animal origin*" or food chain* or food product? or food source* or (food? adj2 (consum* or cooked or importation* or imported or retail* or sample? or uncooked)) or buffet? or marinades or marinated or pickle? or preserved or "ready-to-eat" or RTE).ti. | 25967 |
| 8 | ("animal-based food*" or "food? of animal origin*" or "product? of animal origin*" or food chain* or food product? or food source* or (food? adj2 (consum* or cooked or importation* or imported or retail* or sample? or uncooked)) or buffet? or marinades or marinated or pickle? or preserved or "ready-to-eat" or RTE).ab. /freq=2 | 39220 |
| 9 | ((food? or foodstuff* or meal or meals or snack*) adj2 (convenience or fermented or pre-chopped or prechopped or pre-cooked or precooked or pre-cut or precut or preheated or pre-heated or pre-packed or prepacked or pre-packaged or prepackaged or prepared or pre-prepared or preprepared or pre-sliced or presliced or processed or shop-bought or shop-prepared or store-bought or store-prepared)).ti. | 2113 |
| 10 | ((food? or foodstuff* or meal or meals or snack*) adj2 (convenience or fermented or pre-chopped or prechopped or pre-cooked or precooked or pre-cut or precut or preheated or pre-heated or pre-packed or prepacked or pre-packaged or prepackaged or prepared or pre-prepared or preprepared or pre-sliced or presliced or processed or shop-bought or shop-prepared or store-bought or store-prepared)).ab. /freq=2 | 2362 |
| 11 | (meat? adj2 (consum* or cured or fresh or ground or mince* or product? or raw or retail* or smoked)).ti. | 3365 |
| 12 | (meat? adj2 (consum* or cured or fresh or ground or mince* or product? or raw or retail* or smoked)).ab. /freq=2 | 4594 |
| 13 | ((egg or eggs) adj2 (commercial* or consum* or eat* or product? or raw or retail* or table)).ti. | 765 |
| 14 | ((egg or eggs) adj2 (commercial* or consum* or eat* or product? or raw or retail* or table)).ab. /freq=2 | 960 |
| 15 | (baloney or boloney or burger* or charcuterie* or chorizo* or "cold meat*" or "cold cut*" or "corned beef" or deli or delis or delicatessen* or frankfurter* or hamburger* or hotdog* or hot dog* or "lunch meat*" or "luncheon meat*" or "meatloaf*" or "meat loaf*" or mortadella or pastrami or pate or pates or pepperoni* or "potted meat*" or polony or rillette* or salami* or sausage* or terrine*).ti. | 3314 |
| 16 | (baloney or boloney or burger* or charcuterie* or chorizo* or "cold meat*" or "cold cut*" or "corned beef" or deli or delis or delicatessen* or frankfurter* or hamburger* or hotdog* or hot dog* or "lunch meat*" or "luncheon meat*" or "meatloaf*" or "meat loaf*" or mortadella or pastrami or pate or pates or pepperoni* or "potted meat*" or polony or rillette* or salami* or sausage* or terrine*).ab. /freq=2 | 3685 |
| 17 | ((beef or chicken* or lamb* or pork or poultry or swine or turkey* or veal) adj2 (consum* or ground or meat? or mince* or product? or raw or retail* or smoked)).ti.  [adj2 changed to adj3 on July 28, 2021 to allow for additional coverage of articles on AMR and meat products] | 4651 |
| 18 | ((beef or chicken* or lamb* or pork or poultry or swine or turkey* or veal) adj2 (consum* or ground or meat? or mince* or product? or raw or retail* or smoked)).ab. /freq=2 | 5641 |
| 19 | (food fish* or (fish* adj2 (consum* or eat* or product? or raw or retail* or smoked))).ti. | 2375 |
| 20 | (food fish* or (fish* adj2 (consum* or eat* or product? or raw or retail* or smoked))).ab. /freq=2 | 3549 |
| 21 | (clam or clams or crab or crabs or crayfish* or lobster? or mackerel? or mussel? or oyster? or prawn? or salmon? or scallop? or "sea food?" or seafood? or shellfish or shrimp* or trout?).ti. | 58525 |
| 22 | (clam or clams or crab or crabs or crayfish* or lobster? or mackerel? or mussel? or oyster? or prawn? or salmon? or scallop? or "sea food?" or seafood? or shellfish or shrimp* or trout?).ab. /freq=2 | 52535 |
| 23 | (bologna or ham or hams or sandwich* or tongue).ti. | 24468 |
| 24 | (bologna or ham or hams or sandwich* or tongue).ab. /freq=2 | 32920 |
| 25 | (fruit? or (fresh adj2 produce) or (processed adj2 produce) or salad? or vegetable? or apple? or apricot* or avocado* or blackberries or blueberries or cantaloupe* or cherries or coconut? or dates or fig or figs or grapes or grapefruit* or kiwifruit* or lemon? or lime or limes or mandarine* or mango or mangoes or melon? or nectarine* or orange* or papaya* or peach* or pears or pineapple* or plum or plums or passion fruit* or raspberries or strawberries or tangerine* or watermelon* or almond* or artichoke* or arugula or asparagus or aubergine* or bak choi or barley or basil or beans or beet green or beetroot* or bok choy or boletus or broccoli* or brussel sprout? or buckwheat or bulgur or butternut squash* or button squash* or cabbage* or carrot? or cassava* or cauliflower* or celeriac* or celeries or celery or chanterelle* or chard? or chestnut* or chickpea* or chicory or cilantro or collard green? or coriander or corn or courgette* or cress or cucumber* or dill or eggplant* or endive* or fennel? or fresh tea or garlic or ginger or hazelnut? or horseradish or kale or kales or leek or leeks or lentil? or lettuce* or lotus root? or macadamia nut? or maize or millet or marjoram or mint or morel or mushroom* or mustard* or nopal or oats or onion? or parsley or parsnip* or pea or peas or peanut? or pepper* or peppermint* or pistachio* or potato or potatoes or pumpkin* or quinoa or radicchio* or radish or radishes or rapini? or rice or rosemary or rutabaga? or rye or sage or shallot? or sorghum or soybean* or spaghetti squash* or spinach or swede or swedes or sweet corn* or sweet potato* or thyme or tomato* or turnip* or triticale or truffle? or yam or yams or yeast extract? or walnut? or wasabi or water chestnut* or watercress or wheat or zucchini*).ti. | 285968 |
| 26 | (fruit? or (fresh adj2 produce) or (processed adj2 produce) or salad? or vegetable? or apple? or apricot* or avocado* or blackberries or blueberries or cantaloupe* or cherries or coconut? or dates or fig or figs or grapes or grapefruit* or kiwifruit* or lemon? or lime or limes or mandarine* or mango or mangoes or melon? or nectarine* or orange* or papaya* or peach* or pears or pineapple* or plum or plums or passion fruit* or raspberries or strawberries or tangerine* or watermelon* or almond* or artichoke* or arugula or asparagus or aubergine* or bak choi or barley or basil or beans or beet green or beetroot* or bok choy or boletus or broccoli* or brussel sprout? or buckwheat or bulgur or butternut squash* or button squash* or cabbage* or carrot? or cassava* or cauliflower* or celeriac* or celeries or celery or chanterelle* or chard? or chestnut* or chickpea* or chicory or cilantro or collard green? or coriander or corn or courgette* or cress or cucumber* or dill or eggplant* or endive* or fennel? or fresh tea or garlic or ginger or hazelnut? or horseradish or kale or kales or leek or leeks or lentil? or lettuce* or lotus root? or macadamia nut? or maize or millet or marjoram or mint or morel or mushroom* or mustard* or nopal or oats or onion? or parsley or parsnip* or pea or peas or peanut? or pepper* or peppermint* or pistachio* or potato or potatoes or pumpkin* or quinoa or radicchio* or radish or radishes or rapini? or rice or rosemary or rutabaga? or rye or sage or shallot? or sorghum or soybean* or spaghetti squash* or spinach or swede or swedes or sweet corn* or sweet potato* or thyme or tomato* or turnip* or triticale or truffle? or yam or yams or yeast extract? or walnut? or wasabi or water chestnut* or watercress or wheat or zucchini*).ab. /freq=2 | 383403 |
| 27 | (consum* or eat* or fresh or importation* or imported or meat? or product? or raw or retail*).tw. | 1995056 |
| 28 | (21 or 22 or 23 or 24 or 25 or 26) and 27 | 135008 |
| 29 | (cheese* or yogo?rt* or (dairy adj2 (beverage? or consum* or drink* or food* or product? or retail*))).ti. | 8832 |
| 30 | (cheese* or yogo?rt* or (dairy adj2 (beverage? or consum* or drink* or food* or product? or retail*))).ab. /freq=2 | 11705 |
| 31 | (milk adj2 (beverage? or consum* or drink* or food* or pasteuri* or product? or raw or unpasteuri*)).ti. | 3897 |
| 32 | (milk adj2 (beverage? or consum* or drink* or food* or pasteuri* or product? or raw or unpasteuri*)).ab. /freq=2 | 5726 |
| 33 | honey.ti. or honey.ab. /freq=2 | 9760 |
| 34 | (consum* or eat* or food* or importation* or imported or raw or retail*).tw. | 1339019 |
| 35 | 33 and 34 | 1881 |
| 36 | 7 or 8 or 9 or 10 or 11 or 12 or 13 or 14 or 15 or 16 or 17 or 18 or 19 or 20 or 28 or 29 or 30 or 31 or 32 or 35 [food] | 227120 |
| 37 | consumer/ or human/ or public health/ | 22337998 |
| 38 | human?.ti. or human?.ab. /freq=2 | 1789885 |
| 39 | (human health or consumer? or "One Health" or people or public health).tw. | 1072763 |
| 40 | 37 or 38 or 39 [human health] | 22798631 |
| 41 | 6 and 36 and 40 [AMR + food + human health] | 1621 |
| 42 | (conference or conference abstract or conference review or editorial or letter or note).pt. | 7618373 |
| 43 | 41 not 42 | 1462 |
| 44 | 41 not 43 | 159 |
| 45 | limit 43 to (yr=2010-current and (english or french or spanish)) [included set] | 1174 |
| 46 | limit 44 to (yr=2010-current and (english or french or spanish)) [excluded set] | 128 |

Database(s): **Ovid MEDLINE(R) ALL**1946 to June 28, 2021
Search Strategy:

| **#** | **Searches** | **Results** |
| --- | --- | --- |
| 1 | Drug Resistance, Microbial/ or exp Drug Resistance, Bacterial/ or Drug Resistance, multiple, Bacterial/ | 150795 |
| 2 | (AMR or ARGs or MRSA or ((antimicrobial* or microbial* or antibacterial* or antibiotic* or anti biotic* or bacteria* or gene or genes or multidrug* or multi drug* or multiple drug*) adj3 resistan*)).ti. | 63856 |
| 3 | (AMR or ARGs or MRSA or ((antimicrobial* or microbial* or antibacterial* or antibiotic* or anti biotic* or bacteria* or gene or genes or multidrug* or multi drug* or multiple drug*) adj3 resistan*)).ab. /freq=2 | 85045 |
| 4 | ((aldesulfone sodium or amidinopenicillin* or amikacin or aminocyclitol or aminoglycoside* or aminopenicillin* or (aminosalicylate* adj2 calcium) or (aminosalicylate* adj2 sodium) or amoxicillin* or amoxicillin-clavulanicacid or amphenicol? or ampicillin* or azidocillin* or ampicillin-sulbactam? or ansamycin? or antipseudomonal? or antistaphylococcal* or apramycin or arbekacin or astromicin or avoparcin or azithromycin or azlocillin* or aztreonam or bacampicillin* or bacitracin* or bedaquilin? or bekanamycin or benethaminebenzylpenicillin* or benzathinebenzylpenicillin* or benzylpenicillin* or besifloxacin or biapenem or brodimoprim or cadazolid or capreomycin or carbapenem? or carbenicillin* or carindacillin* or carumonam or cefacetrile or cefaclor or cefadroxil or cephalexin? or cefalonium or cefaloridin? or cefalothin* or cefalotin* or cefamandole or cefapirin or cefatrizine or cefazedone or cefazolin or cefbuperazone or cefcapene or cefdinir or cefditoren or cefepime or cefetamet or cefixime or cefmenoxime or cefmetazole or cefminox or cefodizime or cefonicid or cefoperazone or cefoperazone-sulbactam or cefoselis or cefotaxime or cefotetan or cefotiam or cefoxitin or cefovecin or cefozopran or ceforanide or cefpiramide or cefpirome or cefpodoxime or cefprozil or cefquinome or cefradine or cefroxadine or cefsulodin or ceftaroline fosamil or ceftazidime or ceftazidime-avibactam or ceftezole or ceftibuten or ceftiofur or ceftizoxime or ceftobiprole or ceftolozane or ceftriaxone or ceftriaxone-sulbactam or cefuroxime or cephalosporin* or cephamycin* or cethromycin or chloramphenicol or chlortetracycline or cinoxacin or ciprofloxacin or clarithromycin or clindamycin or clofazimine or clometocillin* or clomocycline or cloxacillin* or colistin or cycloserine or dalbavancin or danofloxacin or dapsone or daptomycin or delafloxacin* or delamanid or dibekacin* or dicloxacillin* or difloxacin* or dihydrostreptomycin* or dirithromycin* or demeclocyclin* or dihydrofolate reductase inhibitor* or doripenem or doxycycline or enoxacin or enrofloxacin or epicillin* or eravacycline or ertapenem or erythromycin or ethambutol or ethionamide or faropenem or fidaxomicin or fleroxacin or flumequine or fluoroquinolone? or flurithromycin or flomoxef or florfenicol or flucloxacillin* or formosulfathiazole or fosfomycin or framycetin or furaltadone or furazidin or furazolidone or fusidane or fusidic acid* or gamithromycin or garenoxacin* or gatifloxacin* or gemifloxacin* or gentamicin* or glycopeptide* or glycylcyclin* or grepafloxacin* or hetacillin* or ibafloxacin or iclaprim or imipenem or isepamicin or isoniazid or josamycin or kanamycin or ketolide? or kitasamycin or latamoxef or levofloxacin or lincomycin or lincosamide? or linezolid or lipoglycopeptide? or lipopeptide? or lomefloxacin or loracarbef or lymecycline or macrolide? or marbofloxacin or mecillinam or meropenem or metampicillin* or mezlocillin* or metacycline or methicillin* or meticillin* or metronidazole or midecamycin or minocycline or miocamycin or monobactam* or morinamide or moxifloxacin or mupirocin or nadifloxacin or nafcillin* or nalidixic acid* or naphcillin* or neomycin or netilmicin or nifurtoinol or nitrofuran? or nitrofurantoin or nitrofural or nitroimidazole* or norfloxacin or ofloxacin or oleandomycin* or omadacyclin* or orbifloxacin or oritavancin or ornidazole or oxacillin* or oxazolidinone? or oxolinic acid* or oxytetracycline or ozenoxacin or panipenem or para-aminosalicylic-acid* or paromomycin or pazufloxacin or pefloxacin or penamecillin* or penethamate hydriodide or penicillin? or penimepicycline or pheneticillin* or phenoxymethylpenicillin* or phthalylsulfathiazole or pipemidic acid* or piperacillin* or piperacillin-tazobactam or pirlimycin or piromidic acid* or pivampicillin* or pivmecillinam or plazomicin or pleuromutilin? or polypeptide? or polymyxin? or polymyxin b or polymixin e or pradofloxacin or pristinamycin or procaine or propicillin* or protionamide or prulifloxacin or pseudomonic acid* or pyrazinamide or pyrimethamine or quinolone? or quinupristin-dalfopristin or ramoplanin or radezolid or retapamulin or ribostamycin or rifabutin or rifampicin or rifamycin or rifapentine or rifaximin or riminofenazine* or rokitamycin or rolitetracycline or rosoxacin or roxithromycin or rufloxacin or secnidazole or sitafloxacin or solithromycin or sparfloxacin or spectinomycin or spiramycin or streptogramin? or streptomycin or sulbenicillin* or sulfadiazine or sulfadimethoxine or sulfadimidine or sulfafurazole or sulfaisodimidine or sulfalene or sulfamazone or sulfamerazine or sulfamethizole or sulfamethoxazole or sulfamethoxypyridazine or sulfametomidine or sulfametoxydiazine or sulfametrole or sulfamoxole or sulfanilamide or sulfaperin or sulfaphenazole or sulfapyridine or sulfathiazole or sulfathiourea or sulfisoxazole or sulfonamide? or sulfone or sulfones or sultamicillin* or talampicillin* or tazobactam or tedizolid or teicoplanin or telavancin or telithromycin or temafloxacin or temocillin* or terizidone or tetracycline? or tetroxoprim or thiamphenicol or tiamulin or ticarcillin* or ticarcillin-clavulanicacid or tigecycline or tildipirosin or tilmicosin or tinidazole or tiocarlide or tobramycin or trimethoprim or troleandomycin or tulathromycin or tylvalosin or tylosin or vaborbactam or valnemulin or vancomycin or virginiamycin) adj2 resistan*).ti. | 43111 |
| 5 | ((aldesulfone sodium or amidinopenicillin* or amikacin or aminocyclitol or aminoglycoside* or aminopenicillin* or (aminosalicylate* adj2 calcium) or (aminosalicylate* adj2 sodium) or amoxicillin* or amoxicillin-clavulanicacid or amphenicol? or ampicillin* or azidocillin* or ampicillin-sulbactam? or ansamycin? or antipseudomonal? or antistaphylococcal* or apramycin or arbekacin or astromicin or avoparcin or azithromycin or azlocillin* or aztreonam or bacampicillin* or bacitracin* or bedaquilin? or bekanamycin or benethaminebenzylpenicillin* or benzathinebenzylpenicillin* or benzylpenicillin* or besifloxacin or biapenem or brodimoprim or cadazolid or capreomycin or carbapenem? or carbenicillin* or carindacillin* or carumonam or cefacetrile or cefaclor or cefadroxil or cephalexin? or cefalonium or cefaloridin? or cefalothin* or cefalotin* or cefamandole or cefapirin or cefatrizine or cefazedone or cefazolin or cefbuperazone or cefcapene or cefdinir or cefditoren or cefepime or cefetamet or cefixime or cefmenoxime or cefmetazole or cefminox or cefodizime or cefonicid or cefoperazone or cefoperazone-sulbactam or cefoselis or cefotaxime or cefotetan or cefotiam or cefoxitin or cefovecin or cefozopran or ceforanide or cefpiramide or cefpirome or cefpodoxime or cefprozil or cefquinome or cefradine or cefroxadine or cefsulodin or ceftaroline fosamil or ceftazidime or ceftazidime-avibactam or ceftezole or ceftibuten or ceftiofur or ceftizoxime or ceftobiprole or ceftolozane or ceftriaxone or ceftriaxone-sulbactam or cefuroxime or cephalosporin* or cephamycin* or cethromycin or chloramphenicol or chlortetracycline or cinoxacin or ciprofloxacin or clarithromycin or clindamycin or clofazimine or clometocillin* or clomocycline or cloxacillin* or colistin or cycloserine or dalbavancin or danofloxacin or dapsone or daptomycin or delafloxacin* or delamanid or dibekacin* or dicloxacillin* or difloxacin* or dihydrostreptomycin* or dirithromycin* or demeclocyclin* or dihydrofolate reductase inhibitor* or doripenem or doxycycline or enoxacin or enrofloxacin or epicillin* or eravacycline or ertapenem or erythromycin or ethambutol or ethionamide or faropenem or fidaxomicin or fleroxacin or flumequine or fluoroquinolone? or flurithromycin or flomoxef or florfenicol or flucloxacillin* or formosulfathiazole or fosfomycin or framycetin or furaltadone or furazidin or furazolidone or fusidane or fusidic acid* or gamithromycin or garenoxacin* or gatifloxacin* or gemifloxacin* or gentamicin* or glycopeptide* or glycylcyclin* or grepafloxacin* or hetacillin* or ibafloxacin or iclaprim or imipenem or isepamicin or isoniazid or josamycin or kanamycin or ketolide? or kitasamycin or latamoxef or levofloxacin or lincomycin or lincosamide? or linezolid or lipoglycopeptide? or lipopeptide? or lomefloxacin or loracarbef or lymecycline or macrolide? or marbofloxacin or mecillinam or meropenem or metampicillin* or mezlocillin* or metacycline or methicillin* or meticillin* or metronidazole or midecamycin or minocycline or miocamycin or monobactam* or morinamide or moxifloxacin or mupirocin or nadifloxacin or nafcillin* or nalidixic acid* or naphcillin* or neomycin or netilmicin or nifurtoinol or nitrofuran? or nitrofurantoin or nitrofural or nitroimidazole* or norfloxacin or ofloxacin or oleandomycin* or omadacyclin* or orbifloxacin or oritavancin or ornidazole or oxacillin* or oxazolidinone? or oxolinic acid* or oxytetracycline or ozenoxacin or panipenem or para-aminosalicylic-acid* or paromomycin or pazufloxacin or pefloxacin or penamecillin* or penethamate hydriodide or penicillin? or penimepicycline or pheneticillin* or phenoxymethylpenicillin* or phthalylsulfathiazole or pipemidic acid* or piperacillin* or piperacillin-tazobactam or pirlimycin or piromidic acid* or pivampicillin* or pivmecillinam or plazomicin or pleuromutilin? or polypeptide? or polymyxin? or polymyxin b or polymixin e or pradofloxacin or pristinamycin or procaine or propicillin* or protionamide or prulifloxacin or pseudomonic acid* or pyrazinamide or pyrimethamine or quinolone? or quinupristin-dalfopristin or ramoplanin or radezolid or retapamulin or ribostamycin or rifabutin or rifampicin or rifamycin or rifapentine or rifaximin or riminofenazine* or rokitamycin or rolitetracycline or rosoxacin or roxithromycin or rufloxacin or secnidazole or sitafloxacin or solithromycin or sparfloxacin or spectinomycin or spiramycin or streptogramin? or streptomycin or sulbenicillin* or sulfadiazine or sulfadimethoxine or sulfadimidine or sulfafurazole or sulfaisodimidine or sulfalene or sulfamazone or sulfamerazine or sulfamethizole or sulfamethoxazole or sulfamethoxypyridazine or sulfametomidine or sulfametoxydiazine or sulfametrole or sulfamoxole or sulfanilamide or sulfaperin or sulfaphenazole or sulfapyridine or sulfathiazole or sulfathiourea or sulfisoxazole or sulfonamide? or sulfone or sulfones or sultamicillin* or talampicillin* or tazobactam or tedizolid or teicoplanin or telavancin or telithromycin or temafloxacin or temocillin* or terizidone or tetracycline? or tetroxoprim or thiamphenicol or tiamulin or ticarcillin* or ticarcillin-clavulanicacid or tigecycline or tildipirosin or tilmicosin or tinidazole or tiocarlide or tobramycin or trimethoprim or troleandomycin or tulathromycin or tylvalosin or tylosin or vaborbactam or valnemulin or vancomycin or virginiamycin) adj2 resistan*).ab. /freq=2 | 43540 |
| 6 | 1 or 2 or 3 or 4 or 5 [AMR] | 229906 |
| 7 | ("animal-based food*" or "food? of animal origin*" or "product? of animal origin*" or food chain* or food product? or food source* or (food? adj2 (consum* or cooked or importation* or imported or retail* or sample? or uncooked)) or buffet? or marinades or marinated or pickle? or preserved or "ready-to-eat" or RTE).ti. | 21924 |
| 8 | ("animal-based food*" or "food? of animal origin*" or "product? of animal origin*" or food chain* or food product? or food source* or (food? adj2 (consum* or cooked or importation* or imported or retail* or sample? or uncooked)) or buffet? or marinades or marinated or pickle? or preserved or "ready-to-eat" or RTE).ab. /freq=2 | 29039 |
| 9 | ((food? or foodstuff* or meal or meals or snack*) adj2 (convenience or fermented or pre-chopped or prechopped or pre-cooked or precooked or pre-cut or precut or preheated or pre-heated or pre-packed or prepacked or pre-packaged or prepackaged or prepared or pre-prepared or preprepared or pre-sliced or presliced or processed or shop-bought or shop-prepared or store-bought or store-prepared)).ti. | 2038 |
| 10 | ((food? or foodstuff* or meal or meals or snack*) adj2 (convenience or fermented or pre-chopped or prechopped or pre-cooked or precooked or pre-cut or precut or preheated or pre-heated or pre-packed or prepacked or pre-packaged or prepackaged or prepared or pre-prepared or preprepared or pre-sliced or presliced or processed or shop-bought or shop-prepared or store-bought or store-prepared)).ab. /freq=2 | 2005 |
| 11 | (meat? adj2 (consum* or cured or fresh or ground or mince* or product? or raw or retail* or smoked)).ti. | 3470 |
| 12 | (meat? adj2 (consum* or cured or fresh or ground or mince* or product? or raw or retail* or smoked)).ab. /freq=2 | 4360 |
| 13 | ((egg or eggs) adj2 (commercial* or consum* or eat* or product? or raw or retail* or table)).ti. | 722 |
| 14 | ((egg or eggs) adj2 (commercial* or consum* or eat* or product? or raw or retail* or table)).ab. /freq=2 | 770 |
| 15 | (baloney or boloney or burger* or charcuterie* or chorizo* or "cold meat*" or "cold cut*" or "corned beef" or deli or delis or delicatessen* or frankfurter* or hamburger* or hotdog* or hot dog* or "lunch meat*" or "luncheon meat*" or "meatloaf*" or "meat loaf*" or mortadella or pastrami or pate or pates or pepperoni* or "potted meat*" or polony or rillette* or salami* or sausage* or terrine*).ti. | 4101 |
| 16 | (baloney or boloney or burger* or charcuterie* or chorizo* or "cold meat*" or "cold cut*" or "corned beef" or deli or delis or delicatessen* or frankfurter* or hamburger* or hotdog* or hot dog* or "lunch meat*" or "luncheon meat*" or "meatloaf*" or "meat loaf*" or mortadella or pastrami or pate or pates or pepperoni* or "potted meat*" or polony or rillette* or salami* or sausage* or terrine*).ab. /freq=2 | 4047 |
| 17 | ((beef or chicken* or lamb* or pork or poultry or swine or turkey* or veal) adj2 (consum* or ground or meat? or mince* or product? or raw or retail* or smoked)).ti.  [adj2 changed to adj3 on July 28, 2021 to allow for additional coverage of articles on AMR and meat products] | 5081 |
| 18 | ((beef or chicken* or lamb* or pork or poultry or swine or turkey* or veal) adj2 (consum* or ground or meat? or mince* or product? or raw or retail* or smoked)).ab. /freq=2 | 5638 |
| 19 | (food fish* or (fish* adj2 (consum* or eat* or product? or raw or retail* or smoked))).ti. | 2158 |
| 20 | (food fish* or (fish* adj2 (consum* or eat* or product? or raw or retail* or smoked))).ab. /freq=2 | 3005 |
| 21 | (clam or clams or crab or crabs or crayfish* or lobster? or mackerel? or mussel? or oyster? or prawn? or salmon? or scallop? or "sea food?" or seafood? or shellfish or shrimp* or trout?).ti. | 56418 |
| 22 | (clam or clams or crab or crabs or crayfish* or lobster? or mackerel? or mussel? or oyster? or prawn? or salmon? or scallop? or "sea food?" or seafood? or shellfish or shrimp* or trout?).ab. /freq=2 | 47562 |
| 23 | (bologna or ham or hams or sandwich* or tongue).ti. | 24734 |
| 24 | (bologna or ham or hams or sandwich* or tongue).ab. /freq=2 | 26450 |
| 25 | (fruit? or (fresh adj2 produce) or (processed adj2 produce) or salad? or vegetable? or apple? or apricot* or avocado* or blackberries or blueberries or cantaloupe* or cherries or coconut? or dates or fig or figs or grapes or grapefruit* or kiwifruit* or lemon? or lime or limes or mandarine* or mango or mangoes or melon? or nectarine* or orange* or papaya* or peach* or pears or pineapple* or plum or plums or passion fruit* or raspberries or strawberries or tangerine* or watermelon* or almond* or artichoke* or arugula or asparagus or aubergine* or bak choi or barley or basil or beans or beet green or beetroot* or bok choy or boletus or broccoli* or brussel sprout? or buckwheat or bulgur or butternut squash* or button squash* or cabbage* or carrot? or cassava* or cauliflower* or celeriac* or celeries or celery or chanterelle* or chard? or chestnut* or chickpea* or chicory or cilantro or collard green? or coriander or corn or courgette* or cress or cucumber* or dill or eggplant* or endive* or fennel? or fresh tea or garlic or ginger or hazelnut? or horseradish or kale or kales or leek or leeks or lentil? or lettuce* or lotus root? or macadamia nut? or maize or millet or marjoram or mint or morel or mushroom* or mustard* or nopal or oats or onion? or parsley or parsnip* or pea or peas or peanut? or pepper* or peppermint* or pistachio* or potato or potatoes or pumpkin* or quinoa or radicchio* or radish or radishes or rapini? or rice or rosemary or rutabaga? or rye or sage or shallot? or sorghum or soybean* or spaghetti squash* or spinach or swede or swedes or sweet corn* or sweet potato* or thyme or tomato* or turnip* or triticale or truffle? or yam or yams or yeast extract? or walnut? or wasabi or water chestnut* or watercress or wheat or zucchini*).ti. | 293116 |
| 26 | (fruit? or (fresh adj2 produce) or (processed adj2 produce) or salad? or vegetable? or apple? or apricot* or avocado* or blackberries or blueberries or cantaloupe* or cherries or coconut? or dates or fig or figs or grapes or grapefruit* or kiwifruit* or lemon? or lime or limes or mandarine* or mango or mangoes or melon? or nectarine* or orange* or papaya* or peach* or pears or pineapple* or plum or plums or passion fruit* or raspberries or strawberries or tangerine* or watermelon* or almond* or artichoke* or arugula or asparagus or aubergine* or bak choi or barley or basil or beans or beet green or beetroot* or bok choy or boletus or broccoli* or brussel sprout? or buckwheat or bulgur or butternut squash* or button squash* or cabbage* or carrot? or cassava* or cauliflower* or celeriac* or celeries or celery or chanterelle* or chard? or chestnut* or chickpea* or chicory or cilantro or collard green? or coriander or corn or courgette* or cress or cucumber* or dill or eggplant* or endive* or fennel? or fresh tea or garlic or ginger or hazelnut? or horseradish or kale or kales or leek or leeks or lentil? or lettuce* or lotus root? or macadamia nut? or maize or millet or marjoram or mint or morel or mushroom* or mustard* or nopal or oats or onion? or parsley or parsnip* or pea or peas or peanut? or pepper* or peppermint* or pistachio* or potato or potatoes or pumpkin* or quinoa or radicchio* or radish or radishes or rapini? or rice or rosemary or rutabaga? or rye or sage or shallot? or sorghum or soybean* or spaghetti squash* or spinach or swede or swedes or sweet corn* or sweet potato* or thyme or tomato* or turnip* or triticale or truffle? or yam or yams or yeast extract? or walnut? or wasabi or water chestnut* or watercress or wheat or zucchini*).ab. /freq=2 | 326606 |
| 27 | (consum* or eat* or fresh or importation* or imported or meat? or product? or raw or retail*).tw. | 1597161 |
| 28 | (21 or 22 or 23 or 24 or 25 or 26) and 27 | 114967 |
| 29 | (cheese* or yogo?rt* or (dairy adj2 (beverage? or consum* or drink* or food* or product? or retail*))).ti. | 8373 |
| 30 | (cheese* or yogo?rt* or (dairy adj2 (beverage? or consum* or drink* or food* or product? or retail*))).ab. /freq=2 | 10092 |
| 31 | (milk adj2 (beverage? or consum* or drink* or food* or pasteuri* or product? or raw or unpasteuri*)).ti. | 3986 |
| 32 | (milk adj2 (beverage? or consum* or drink* or food* or pasteuri* or product? or raw or unpasteuri*)).ab. /freq=2 | 4905 |
| 33 | honey.ti. or honey.ab. /freq=2 | 8832 |
| 34 | (consum* or eat* or food* or importation* or imported or raw or retail*).tw. | 1071754 |
| 35 | 33 and 34 | 1624 |
| 36 | 7 or 8 or 9 or 10 or 11 or 12 or 13 or 14 or 15 or 16 or 17 or 18 or 19 or 20 or 28 or 29 or 30 or 31 or 32 or 35 [food] | 194362 |
| 37 | exp Age Groups/ or Humans/ or Public Health/ | 19469945 |
| 38 | human?.ti. or human?.ab. /freq=2 | 1541673 |
| 39 | (human health or consumer? or "One Health" or people or public health).tw. | 846457 |
| 40 | 37 or 38 or 39 [human health] | 19894216 |
| 41 | 6 and 36 and 40 [AMR + food + human health] | 1708 |
| 42 | (comment or congress or editorial or letter or news).pt. | 2226918 |
| 43 | 41 not 42 | 1684 |
| 44 | 41 not 43 | 24 |
| 45 | limit 43 to (yr=2010-current and (english or french or spanish)) [included set] | 1267 |
| 46 | limit 44 to (yr=2010-current and (english or french or spanish)) [excluded set] | 12 |
